# Timing structures in live comedy: A matched-sequence approach to mapping performance dynamics

**DOI:** 10.1093/pnasnexus/pgaf394

**Published:** 2026-01-20

**Authors:** Vanessa C Pope, Rebecca Stewart, Elaine Chew

**Affiliations:** Department of Engineering and School of Biomedical Engineering & Imaging Sciences, King’s College London, London WC2R 2LS, United Kingdom; Dyson School of Design Engineering, Imperial College London, London SW7 2AZ, United Kingdom; Department of Engineering and School of Biomedical Engineering & Imaging Sciences, King’s College London, London WC2R 2LS, United Kingdom

**Keywords:** live performance, informatics, comedy, speech, cultural analytics

## Abstract

Live performance is a ubiquitous cultural and social behavior that has not yet benefited from systematic scientific study. We present a computational methodology that visualizes and describes timing structures in live performance, showcasing their engineering. This novel analysis framework, Topology Analysis of Matching Sequences (TAMS), automatically detects matching sequences and maps their timing. Locating material that is repeated across performances reveals the skill behind apparently effortless communication between performer and audience. Applying TAMS to two stand-up comedy tours uncovered structural features at the macro- and microlevels, including consistently placed novel material at the beginning of shows and sections dedicated to tightly timed repeated material. TAMS also provides a new frame of reference for examining audience–performer dynamics through speech microtiming and laughter. TAMS can be applied to other forms of repeated speech, such as political stump speeches, as well as extended to other types of performance, such as dance.

Significance Statement Scientific frameworks that describe a performance’s engineering may help protect the intellectual property and livelihood of performing arts professionals, now under threat from generative AI. The value we place on human artistry in the performing arts is under question and commercial pressure, particularly in recorded performance. Without tools to visualize live performance’s complexity, we underestimate, mystify, and devalue the skills of performing arts professionals. Live performance is an interplay between rehearsed expressive material and spontaneous adaptation. While joke order in a comedy routine may be largely the same every night, responding to a heckler may not be. Mapping the timing of repeated material across live stand-up comedy performances highlights performers’ skill in enacting flexible communication structures.

## Introduction

Performance—dance, music, song and storytelling—is a cultural feature worldwide, from wayang kulit puppetry in Indonesia to annual pantomimes in the United Kingdom. In the global North, music tours sell out, new shows open and the cultural economy of live performance remains strong despite the rise of recorded media (1, 2). While designed to look spontaneous to an audience that sees only one performance, a show has been carefully structured to allow enough variability to be responsive without compromising content or repeatability. Without the tools to represent the dynamic processes that culminate in a given performance, it’s easy to forget the creative and technical skill involved in their creation. In an output-driven economy, this lack of understanding puts performing arts professionals’ livelihoods at risk. In 2023, it took a 118 day strike to protect scriptwriters’ work from AI, and video game actors went on strike again over the use of AI in 2024 (3, 4). Developing a new framework for understanding the complexity of performance dynamics is crucial at a time when those working in the performing arts are increasingly under pressure to compete with generative AI.

### Performance dynamics in speech

Using AI to generate expressive performance may lead to media artifacts that are credible interpretations, if taken in isolation. But the idea that skilled performance lies in generating a single correct, best or optimal expression is deeply flawed and threatens performers’ careers as well as the diversity of our cultural landscape. Live performance depends on the interaction between the prepared structure of a show and its live responsiveness. A practiced performer with good material is able to navigate the two in real-time to maintain connection with each audience. With a strong bond between audience, performer and material, a live performance can play with the boundaries of credibility rather than be limited by them. Expressive live performance can stretch audience members’ imaginations and experiences in a way that media generated to feel credible cannot.

Increasingly, generative AI research and headlines tempt us to collapse the complexity of the arts into stand-alone, high-scoring, compelling instances and artefacts. A new representation of live performance is required to make performer skill visible. To analyze and appreciate the dynamic, interactive nature of live performance, performance content and timing needs to be compared across multiple performances. In this article, we present a computational, cultural analytics approach that relies tracking performance time and variability. By highlighting patterns across performances, we shift the paradigm away from performance-as-artifact to performance-as-dynamic to reveal the underlying structure of a show.

Even scripted performance adapts to audiences. One of the distinguishing features of performed speech is that it is spoken for an audience and designed to accommodate their external or imagined internal response. Theatre director Peter Brook described how his cast varied their delivery of King Lear during a world tour in 1962 in response to audiences (5). One audience “affected the actors as though a brilliant light were turned on in their work,” while the cast exploited “every bit of exciting action or […] burst of melodrama” (p. 22) for another, and in a cavernous auditorium “they faced the front, spoke loudly and quite rightly threw away all that had become precious about their work” (p. 23). Because of the responsiveness expected of the audience, stand-up comedy is “perhaps the most vulnerable of stage forms. If the jokes don’t go down with the audience, the performer may be literally laughed—or ‘silenced’—off the stage” (p. 563) (6).

### Stand-up comedy as data

Stand-up comedy can freely vary its content, from moving a pause to adding a joke to interacting with the audience. Professional comedians are experts in their craft, paid to use performed speech to entertain and give audiences the thrill of responsive immediacy. But comedians do repeat jokes and tour the same show to different venues: part of their job is creating a similar “spontaneous” experience for each audience. Some material stays the same between shows, despite the infinite variation possible. A professional comedian, after pitching new material to many different audiences, re-uses joke phrasing that meets their communicative needs. Exactly repeated sequences of material are kept in a show because they are functional. Sequences of words and hesitations survive in the evolving structure of a show because they fulfil the performer’s goal. When, how, and to what extent repeated sequences persevere between shows illustrates where the performer adapts their material for different audiences and where they stick to familiar phrasing. As within an evolving organism, functional features are more likely to survive each performance.

Stand-up comedians are generally both creators and interpreters of structure, carefully arranging material for live performance. While stand-up comedians may also be writers, their craft is primarily oral and responsive. Like most performers, comedians tailor delivery to live audiences. A stand-up comedy show is developed over time through testing jokes, altering wording and the orderings of phrases. Timing is a crucial element to understanding how comedians not only deliver their words but structure their material over the course of a show. Performers create a robust show through practice. Visualizing performers’ reuse and timing of material captures some of the dynamics of their oral craft. Qualitative work in sociology and performance studies has described the nuances of stand-up comedy audience reception and performer variation (7–12), while cultural analytics and digital humanities have shed light on the complex eco-systems surrounding live performance, from its patterns of touring to geographical spread to cast composition (13, 14). Ephemera relating to a performance, such as programmes, or its creation, such as design sketches, are often the object of research in computational performance studies. However, other data types are increasingly available, such as YouTube videos of performances, and would benefit from a computational methodology to support their analysis. For example, a study tracing how cast members changed during a show’s 2-year tour could be paired with an analysis of how show structure, content and timing interacted with cast members coming and going. More and more, we have access to multiple recordings of the same show, and with improved database and archive connectivity, of multiple editions of a script.

### Performance dynamics as data: TAMS

So far, most computational approaches to performance prioritize the analysis and interpretation of content and context rather than performance dynamics. Script and motion data have been subject to empirical analysis (15, 16). Often, performance content data are treated as descriptive of the performance-as-artifact rather than as variables in dynamic systems, though the comparison of scripts and text evolution over time move closer to this paradigm. Alignment between dramatic texts has been used to compare translations of Shakespeare’s *Othello* using bespoke visualization tools (17, 18) and text reuse detection looks for patterns across texts, particularly across historical corpora (19, 20). While these analyses do not consider performance timing, their visualizations offer the same affordance of highlighting structural change and ordering as the work presented here.

In this article, we present an algorithm-assisted method for the analysis of live performance to complement and augment current computational and qualitative approaches. By examining repeated performances, we leverage the form’s responsive, repeatable and time-bound nature to uncover its structures and dynamics. Each performance is treated as an expression variant of a show, rather than a stable artifact. We visualize matching sequences of performance content (in this instance, speech) in performance time across multiple performances. This simple approach, inspired by genetics and music performance research, clarifies what frequently appearing sequences are likely to be crucial to a show and how performers express these sequences in performance time. Mapping matching sequences also makes sections of variability visible, pointing to parts of a performance where audience context may be more critical than content. To showcase this methodology, Topology Analysis of Matching Sequences (TAMS), we have applied it to two stand-up comedians on tour. Our case-studies demonstrate how TAMS can be applied to macro and microstructures in stand-up comedy, and to both its generation and reception.

The methodology relies on the shared importance of expressive timing in music and speech, making it possible to apply existing methods from music performance analysis to performed speech. Analysis of performance content has not been subject to the same range of empirical approaches as audience perception, on which psychology, cognitive science, and neuroscience have all been applied (21). In contrast, comparing expressive patterns to discuss structure is an established technique in musical performance studies. The idea of studying multiple performances to uncover rhythmic nuance is established in music performance analysis, where notable musicians’ and pieces’ expressive timing has been compared over large datasets and in the lab (22–28).

Structures identified in musical performance features can be cross-referenced to the structures visible in a score, a check almost impossible when working with performed speech. Unlike a musical score, a script contains little information on rhythmic delivery. Some aspects of rhythm can be thought of as encoded in the words of the script, as they are in musical notes; some words are longer than others, which inherently affects where pauses are likely to be. The duration of words and pauses clarifies ambiguous utterances (29) and content can impact pause duration (30). While there are functional parallels between script and score, the dynamics of a performance are more open to interpretation in a script.

As musicians do, performers control the length of silences and vary tempo to indicate structure and dramatic tension. Timing interacts with other dynamics. In music, a loud note needs time to dissipate before the next can be heard: loudness affects timing (31) and reverberations in a performance space affect how quickly notes are played (32). Performance timing, in speech as in music, is not only a performance dynamic in its own right, but contains traces of other performance factors such as venue acoustics. Timing information encompasses elements of context as well as expressive intent. Speech timing and prosody, its melody, can be analyzed using musical frameworks. Strong links between rhythms in language and rhythms in music have been identified (33–35), and timing and prosody in speech help to structure it (36). Timing in language, as in music, is manipulated to help convey meaning, with speakers elongating pauses to emphasize syntactic boundaries (37) and elongating key words in ambiguous sentences to aid the listener’s comprehension (38).

The TAMS methodology makes few assumptions about what elements of a performance are its key variables. In the following case studies, a word or word-like sound is considered the smallest possible unit, and sequences of six units (or tokens) are the preset minimum used by the algorithm to make sure meaningful sequences are chosen. The primary performative variation we examine is the timing and consistency of performer’s words and word-like sounds, captured in detailed transcripts. The variation in wording inherent to stand-up comedy allows repeated sequences themselves to become units for macro and microscale timing pattern analysis.

When do matched sequences occur in performance time? Is their placement consistent, revealing structure in repeated material? How does the content and timing of jokes evolve through their development? How does performance timing interact with real-time audience response?

Dense clusters of matched sequences form content pillars, highlighting structurally important sections that vary minimally in content. Systematic variability can be distinguished from random variation by its consistency. Error and context-driven variation are randomly distributed, but performance-time with no repeated material in any show is likely to be crucial to the show as a section tailored to each performance’s audience. TAMS allows us to examine structure in expressive timing, too, by comparing the relative timing of matched sequences in different performances. If multiple pairs of matched sequences appear at similar offsets, they become visible as a timing beam, a chain of matched sequences expressed with consistent relative timing across performances.

First, we will present TAMS analyses that highlight macrostructures visible content pillars and timing beams, and how their placement differs in two stand-up comedy tours. In a comedy show in development, TAMS visualizations reveal how show content accumulated over performance iterations, shedding light on how a stand-up comedy show evolved and stabilized over time. Second, we use TAMS analysis to look more closely at the timing dynamics of audience response and performer speech. We find patterns in audience silence, and are able to trace the evolution of the timing of a joke across 20 performances, before demonstrating how audience impacts the microtiming of a joke.

## Results

### Macrostructures: content pillars, timing beams, and show construction

#### Matching sequences forming content pillars

To understand how professional comedians present the same material across performances, the first step is to identify how much content is repeated and in what ways. Transcripts from multiple performances by two stand-up comedians, known in this article as comedian A and comedian B, are analyzed using the computational methodology of sequence matching developed for this research. The two performers’ shows differ in level of show development, performer experience and timescale of data collection, providing a sense of how external factors might impact repeating content. Both professional stand-up comedians used hesitation sounds and apparent errors as part of their recurring delivery, that is, within matching sequences. A matching sequence is a contiguous sequence of tokens, in this case words or hesitation sounds, that is exactly repeated in two shows. Comparing transcripts of performances to the source text shows how a comedian moves from text to speech and how they modify their own text when performing to make it appear conversational rather than literary.

For experienced performer comedian A with an established touring show, an average of 39.66% of each transcript exactly matched to another show’s (see Methods for more detail on algorithm). By contrast, an average of only 10.50% of each transcript was matched to sequences from the script (9.75–10.98%), though this is in part due to the difference in style between text and speech. Most material extracted from the script is presented in a defined order, with 98.07% of the matches in sequential order.

For comedian B, an emerging performer developing a new show, show comparisons found only 14.22% of one transcript matching to another on average. The low average transcript match rate for comedian B is due in large part to the scarcity of matches between the first show recorded and all subsequent performances. Only 1.24% of the performance transcript of 11Aug2018 matched to that of 3Feb2018, demonstrating how much comedian B’s show evolved over the seven months between the first recorded performance in London and the first recorded performance in Edinburgh. The large difference in the first and last of comedian B’s performance naturally impacts all other descriptive statistics, skewing the findings. Regardless of its evolution, the maximum proportion of a transcript matching another within comedian B’s performances was only 34.33% between the two last recorded performances (2018 Aug 17 and 2018 Aug 26), compared to comedian A’s maximum proportion of 51.63%.

**Fig. 1.**
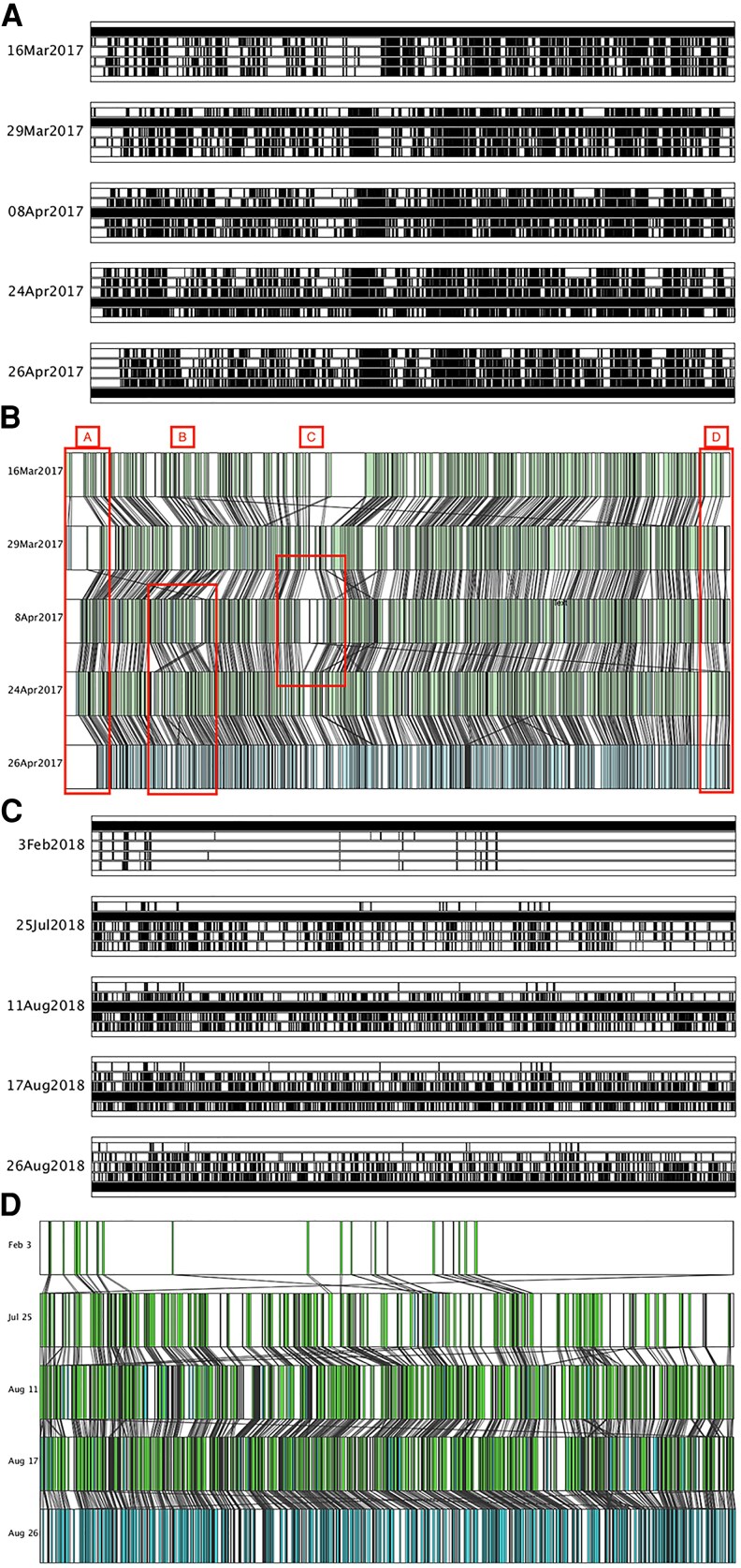
Visualizations of matching sequences for established comedian A (Fig. 1A and B) and emerging comedian B (Fig. 1C and D). Permutation visualizations (Fig. 1A and C) show all matching sequences from all pairs of shows, while chronological visualizations (Fig. 1B and D) show matching sequences in two subsequent shows, tracking how a matching sequence is retained. Comedian A’s established tour has dense clusters of material consistent in all shows, while comedian B’s material builds up over time. A) Matching sequences between all five comedian A shows in each show’s normalized time. B) Matching sequences in chronological pairs of shows in five comedian A performances in each show’s normalized time. C) Matching sequences between all five comedian B shows in each show’s normalized time. D) Matching sequences in chronological pairs of shows in five comedian B performances.

Sequence matching on performance transcripts identified units with which to analyze relative performance timing at a large scale without needing an abstract representation of structure such as a score or a script. Using only performance transcripts, mapping matching sequences clearly illustrates the underlying patterns in performance material as columns of densely matched content, while some sections are consistently blank with improvised, show-specific material (Fig. 1A).

Sequence matching and its accompanying visualizations demonstrate how comedians craft their shows to create a best fit of material for their audiences. Not all comedians use the same tools or manage timing in the same way, as evidenced by the differences in temporal structure between established performer comedian A and emerging performer comedian B. With only two performers analyzed, it’s impossible to say if comedian A’s more evident temporal structure is the result of their expertise or a difference in performance style. Further analysis of the temporal structure of emerging and established comedians would clarify this.

Analysis of the comedy show in development suggests material grows around successful material, which largely stays in the same part of a show (Fig. 1C). We see the number of sequence matches, their duration and length grow over time to become more similar to the metrics of a long-established performance. Not all comedians use the same tools or manage timing in the same way, as evidenced by the differences in temporal structure between established performer comedian A and emerging performer comedian B. Matching sequences can provide information on how rehearsed a show is and potentially how robust it is. TAMS visualizations show how comedians craft their shows to create a best fit of material for their audiences.

#### Show evolution: order and pacing

Both performers have a low percentage of matching sequences that are out of order, referred to here as cross matches as they cut across other pairings (comedian A: 3.56%, comedian B: 5.72%). Cross matches are shorter in length and duration on average than exact sequences (Table 1). The difference is more marked for comedian A than comedian B. Comedian B has more nonsequential matches than comedian A. There are a higher proportion of cross matches before comedian B’s show settles into its run in Edinburgh (comedian B London: 12%; comedian B Edinburgh: 4.63%; comedian A: 3.56%).

**Table 1.** The small proportion of matching sequences (matches) that are out of sequential order (cross match) are shorter than those that are sequential (seq. match).

Cross match	Tokens (mean)	Duration (mean)	Duration (max)
Comedian A	7.60	4.98 s	12.15 s
Comedian B	7.27	4.5 s	10.6 s

Seq. match	Tokens (mean)	Duration (mean)	Duration (max)
Comedian A	12.43	7.00 s	33.12 s
Comedian B	9.43	6.07 s	19.48 s

**Fig. 2.**
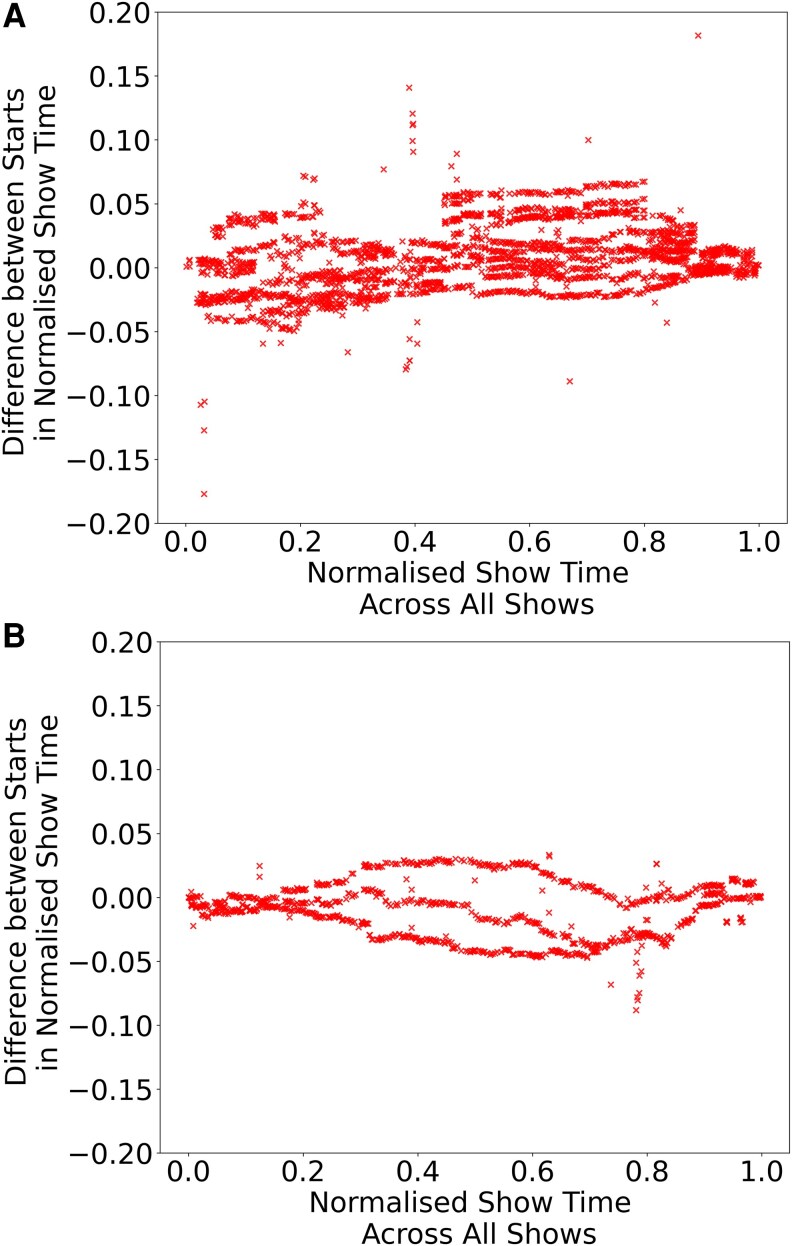
Timing consistency in successive matching sequences, all performances combined. Timing beams are more robust in comedian A’s shows (Fig. 2A) than in comedian B’s shows (Fig. 2B). Matching sequences in different performances appear at more consistent lags in normalized performance time during comedian A’s performances. A) Timing differences between matches in five comedian A performances. B) Timing differences between matches in three comedian B Edinburgh performances.

Lines linking matching sequences, shown in Fig. 2, allow us to see where matches appear in a different order from one show to the other. Crossed lines indicate matching material was presented out of order. In comedian A’s shows this is a rare occurrence as matching material was overwhelming presented in sequential order (Fig. 1B). However, there is a section of material in which two crossed lines appear in similar places between shows 2 and 3 and shows 3 and 4, as well one large cross between shows 4 and 5 (Fig. 1B, just after section C). The cross-matching sequences are not in fact material out of order, but instead a short phrase that is reused twice within that area of material (“want to be a good person”). The phrase “I want to be a good person” occurs twice in different places in all of comedian A’s shows, bookending a segment and explicitly motivating the creation of the show. In show 3, comedian A says sincerely to a quiet audience “actually, here’s my actual question: can you be a good person if you have done bad things? Cos the thing is.” Someone in the audience responds and comedian A laughs, responding: “oh yeah thank you. The show’s finished! I got my answer!” The comedian’s desire to be a better person is presented as the main narrative motivator for the show, the internal repetition of phrases can draw attention to material key to a show’s overall meaning.

The lines drawn between matches provide other information on relative timing: matches of similar length presented at similar intervals draw parallel lines. In all performances there are small blank sections where there were no matches to any of the other performances. Both the chronological and genetic visualization of transcripts have few matches at the very beginning of the show (Fig. 1A and B). The early part of comedian A’s stand-up comedy performance contains little in the way of repeated material. This section of unique material is consistent with the traditional format of stand-up comedy: performers open a show by referencing the venue or recent events before beginning their prepared material. Matching sequences in this part of the performance would be more likely to be standard phrases, for example thanking the audience for their applause.

The genetic visualization of comedian B’s material shows how matching sequences accumulate over time as well as being introduced in chunks (Fig. 1C). Very little of the material in the first show is kept in later shows. The matches between all shows and show 1, visible at the top of the visualization, are sparse. The matches between the last show and previous shows, drawn at the bottom of the visualization, show how the matches accumulated over time. The material does not appear in sections, as you might expect if sections were being written and added in. Instead early material that reappears in the next show is surrounded by more matches in the next show and so on, creating triangular shapes in across the bars representing each performance. Material that is kept in a show seems to act as a kind of seed around which new material is built. Over time, reused material becomes surrounded by other matches that preface it and transition to the next point.

Clusters of parallel lines show exactly repeated material presented at a very similar pace in two consecutive performances (e.g. between sections B and C in Fig. 1B). Abrupt changes in the direction of these connecting lines indicate that material is present in one show that is not in the other, creating a gap between matches (e.g. C in Fig. 1B). If a gap is blank in the second show but has been closed by the third, it indicates an unexpected addition to the show that was later dropped. A connecting line can diverge from the previous one to form a triangular shape that is then filled with new material, integrated into following shows with new parallel lines (e.g. C in Fig. 1B). The unscripted material added to show 3 then matched in shows 4 and 5, showing how additions become embedded over time. All shows have a consistent gap at the start of the show (A in Fig. 1B) and shows 1, 3, 4, and 5 have a consistent gap appearing at 90% of performance time (D in Fig. 1B). Cross-referencing to transcript-to-transcript visualizations at the same point (Fig. 1A) clarifies that shows 1, 2, and 4 share a short sequence at the start of the show, which is not visible in chronological visualizations that only take into account matches between consecutive shows.

Comedian B’s performances were recorded over several months, from the beginning of February to the end of August 2018. Only 25 matches are made between the first and second show, while there is an average of 367 matches per show in consecutive Edinburgh performances. The longest match more than doubles between London and Edinburgh performances (comedian B London: 14, comedian B Edinburgh: 35).

Once the show is in Edinburgh, comedian B’s matched sequences become longer on average, though not as long as comedian A’s (Table 2). Despite sequences being shorter in London, the average time per match is longer (comedian B Lon: 7.09 s, comedian B: Ed: 5.92 s). The maximum time for a match is similar in London and Edinburgh (comedian B Lon: 19 s, comedian B Ed: 19.48 s), despite the maximum length of a sequence in London being less than half that of Edinburgh (comedian B Lon: 14, comedian B Ed: 35). When Edinburgh pairings are considered alone, they have a similar frequency to comedian A’s show (comedian B Ed: 6.17 matches per minute, comedian A: 6.7 matches per minute). The first two shows skew averages for the rest of the material. When the two London shows are removed from the analysis, comedian B’s established show has a similar profile to comedian A’s. In comedian B’s early performances, matched material takes longer to perform, perhaps because of comedian B’s speech is slower or because of audience interaction or response. Audio clips of the matches show that comedian B’s earlier performances have more gaps in speech, both between phrases but also for audience response. The timing of planned material becomes tighter as the show evolves.

**Table 2.** Descriptive statistics of matched sequences found across five comedian A shows (comedian A), five comedian B shows (comedian B), and the three comedian B shows in Edinburgh (comedian BEd).

	Mean	SD	Median	Range
Matches/show				
Comedian A	269.4	34.05	271.5	229–331
Comedian B	180	150.09	181.5	15–396
Comedian BEd	367	46.13	371	319–411
Tokens/match				
Comedian A	12.26	7.98	9	6–72
Comedian B	9.31	4.30	8	6–35
Comedian BEd	9.66	4.66	8	6–35
Match duration				
Comedian A	6.92 s	3.60 s	5.66 s	0–33.12 s
Comedian B	5.98 s	2.87 s	5.64 s	0.80–19.48 s
Comedian BEd	5.92 s	2.76 s	5.60 s	1–19.48 s
Gap btwn matches				
Comedian A	5.86 s	10.23 s	2.44 s	0–69.31 s
Comedian B	76.55 s	131.35 s	21.28 s	0–1,388.2 s
Comedian BEd	6.68 s	8.47 s	3.86 s	0–65.12 s
% show matched				
Comedian A	39.66%	5.45%	39.38%	31.69 –51.63%
Comedian B	14.22%	12.31%	15.10%	1.24–34.33%
Comedian BEd	29.83%	4.08%	30.54%	24.74–34.33%

### Timing beams

Another way of examining timing between shows is to plot the difference between the start times of a match in each show in normalized time (Fig. 2). For example, if a matched sequence begins at 0.25 in show 1 and 0.27 in show 2, the gap in normalized time is 0.02. An offset of zero would mean that a matching sequence occurred at exactly the same point in normalized performance time in both shows. Because every performance has a slightly different duration, matches would not necessarily have the same timestamp but if timed consistently would have similar normalized times. Comedian A’s performances were roughly 40 min long, so an offset of 0.01 represents roughly 20 s in real time. In comedian B’s 60 min shows, a 0.01 offset is ∼36 s.

The offsets between matches in comedian A’s shows behave differently in each section of comedian A’s show. The graph only displays offsets in the −0.15 to 0.15 range, representing ±15% of performance time or offsets between matches, equivalent to ±(40/100)×15=6min in comedian A’s shows. As both the *x* axis and *y* axis share the same unit, normalized performance time, for clarity the *x* axis is expressed as a percentage of performance time and the *y* axis as its 0–1 normalized value. For comedian A, from the start of the show to 20% of the way through, offsets are loosely clustered in a cloud with offsets primarily in the range of −0.05 to 0.05 of normalized time (Fig. 2A). Abruptly, the range of offsets seems to halve to −0.025 to 0.025 until just before the halfway mark. There is also a vertical line of unusually large offsets at the 40% mark in performance time, just before a stretch of similarly timed “beams” of content forming straight stripes across the graph.

Matches that follow quickly one after the other in two shows have similar offsets. The parallel connecting lines of chained matches in chronological visualizations (Fig. 2B) are visible as horizontal lines in a plot of the differences between start times of matching sequences. From halfway through the shows until 80% of the way through, the offsets are mapped in horizontal streaks over a wide range (roughly −0.25 to 0.07). At the very end of the performance, the offsets converge towards zero in three steps: first the streaks abruptly narrow in range (−0.02--0.03), then briefly become two tight clusters between −0.01--0.01 before ending on a tight vertical cluster around zero.

Based on comedian A’s shows running to ∼40 min, the first eight minutes of material have relatively variable timing. The timing then tightens up, with material being performed at much more similar points in the performance. The offsets between matching material drift towards zero before the chained matches are launched halfway through the performance. For about 12 min, matches are presented one after the other as the planned material proceeds smoothly from whatever offset it started with. Then in the final 8 min of the performance, matches begin to converge towards zero again as the performer makes up time. Late or early material is then compensated for as the matches become increasingly synchronized in normalized time. Timing is treated differently in each section of performance: sometimes pacing between matches takes priority, as in sections full of horizontal lines, and in others the overall pacing takes priority, where the offsets between matches converge towards zero. It is remarkable that timing in these sections is manipulated in similar ways in all performances, suggesting these timing dynamics are part of the performance design.

Plotting differences of the matching sequences found in comedian B’s performances in normalized time reveals a looser structure (Fig. 2B). There are noticeably more differences below the zero mark than above. The points plotted are the product of the normalized time in the second show in a match minus the normalized time of the first. The second show in a matched pair is always chronologically later than the first. Comedian B’s show is tightening up over time: matching sequences tended to occur later in normalized performance time in the first show than in the second, creating the negative values on the y axis. Matched sequences were occurring earlier in the show over time, suggesting that the show was becoming more compact as it developed. At the beginning of the show, up to roughly 20% of the show or 12 min in performance time, sequence matches are relatively synchronized with their difference hovering around zero. However, in the middle section, between 20 and 80%, differences in synchronization are scattered. Instead of horizontal lines that suggest clusters of material occurring at the same pace, we see arcs and wobbly lines. Because the graph shows the sequences for all 10 matches, it’s unclear whether these arcs do show the pattern for only one pair of shows. When the offsets between matches are visualized by show, waves in the relative timing of matching sequences are clear in comedian B’s matches where in comedian A’s shows there are more vertical lines, with vertical variations happening in sudden breaks rather than drifts.

### Microstructures: joke development and microtiming parameters

#### Joke development: connecting laughter patterns to TAMS

**Fig. 3.**
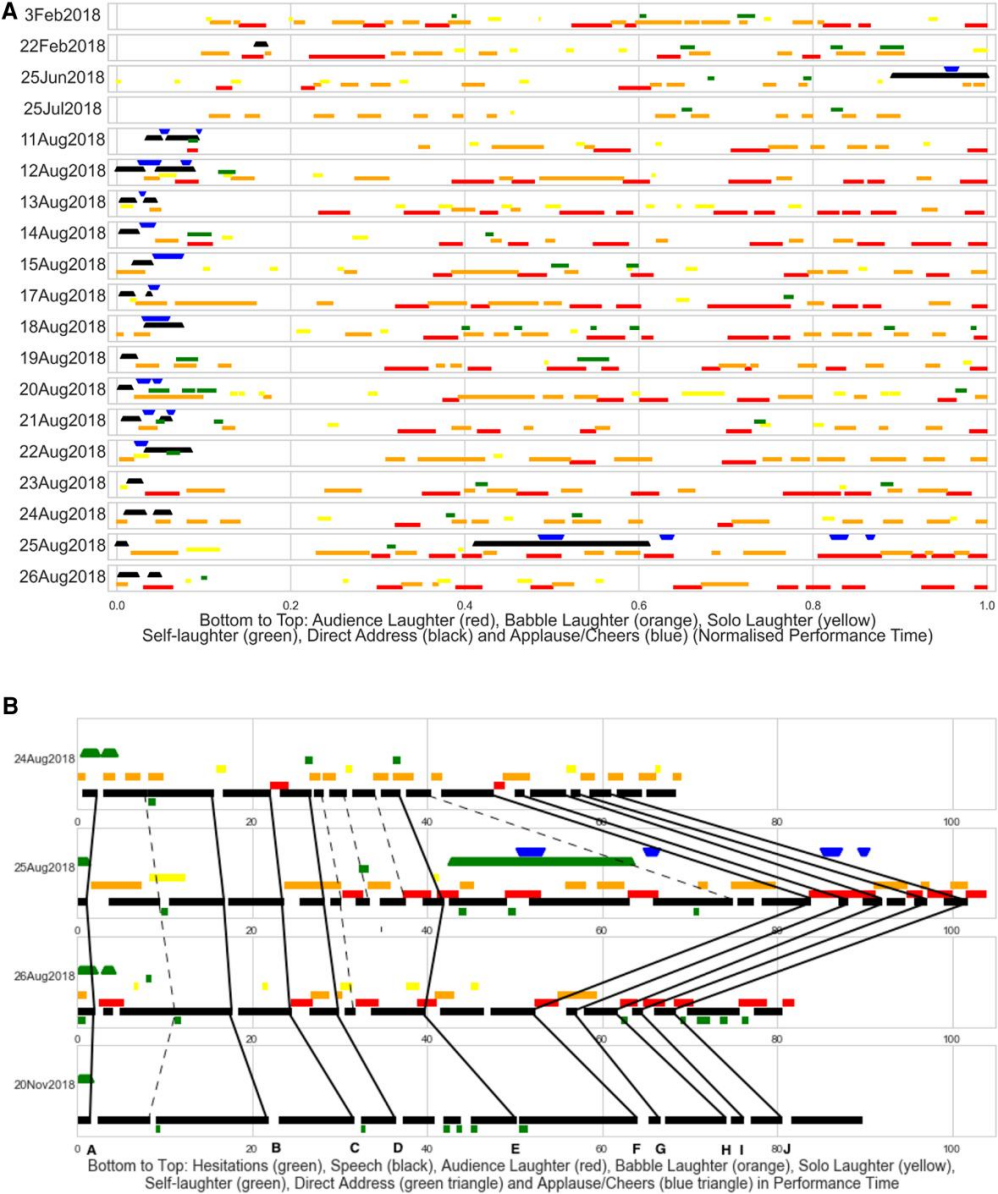
Audience and performer laughter, live and in the lab. A) Audience and performer laughter across 20 tellings of the same joke by comedian B in normalized time. B) Speech segments (black) alongside performer and audience laughter live (top three rows) and in the lab (bottom row) in performance time (seconds). Parallel lines between rows in Fig. 3B indicate greater consistency in timing.

In previous work, we described how patterns in silence are more reliable than patterns in audience laughter in an established professional comedy show (39). Comedian B’s show evolved a great deal in the time it was studied. Only 20 sequences from the first show were found in the last recorded live performance, 2018 Aug 26. Of these 20 sequences, five are part of a joke about Scooby Doo. The Scooby Doo joke contains the maximum number of sequence matches for any one joke and those matches occurred in the smallest timeframe. The Scooby Doo clips varied between 64.22 and 103.81 seconds in length (mean: 78.56 s; SD: 9.95 s). On average 43.14% of the joke’s performance time contained laughter. The proportions of laughter varied between 23.22% (2018 Jul 25) and 62.97% (2018 Aug 25) (Fig. 3A).

The joke is placed after introductory material that touches on niche colonial history references. Comedian B plays at having lost the audience with nerdy history material, returning to the safe common ground of Scooby Doo to keep the audience engaged. In all performances the joke is roughly 5 min into the show. There are three joke endings in the recordings. In the majority of performances, the joke ends when comedian B remarks on the absurdity of casting hippies as auditors, particularly hippies who arrive in town “just as the crimes began.” Fifteen of the 20 clips end with the words “crimes began.” In three of the 20 recordings, the joke ends on an earlier punchline about people “trusting hippies with their finances.” In the performances of 2018 Feb 22 and 2018 Jun 25, comedian B did not use either phrasing. These clips ended when comedian B moved on to the next joke.

There are patterns in the timing of audience response despite differences in scale and type. The earlier London shows have a distinct temporal profile in comparison to those in Edinburgh. The first two performances (Feb 3 and Feb 22) have remarkably similar laughter timing in the first half of the joke: audiences laughter starts a few seconds into the joke, then tails off about halfway through. On 2018 Feb 3, they resume laughing with a long Audience laugh then laugh regularly, whereas on 2018 Feb 22 laughter resumes later with shorter, more sporadic laughter. Although the content of the joke is very similar for the first section of joke, it diverges in the second half. The second set of London performances have less full audience laughter, with more regular laughter from solo audience members or small groups. In Edinburgh performances, only Babble and Solo laughter occur between the introductory section and 0.3 of performance time, except for a short burst of Audience Laughter in 2018 Aug 13. The middle column of Edinburgh shows is dense with types of laughter, including Self-laughter from the performer. In most shows there is a short lull, followed by a burst of audience

The final three live performances were then compared to a recording made in the lab a few months later. Ten pauses present in all four performances are preceded by closely matched phrases. Nine of these matches are exact, except for the last where comedian B used synonyms (“financial matters”, “financial affairs” and “finances” chronologically at point J in Fig. 3B). In two of the shows, 2018 Aug 25 and 2018 Nov 20, comedian B pauses around a hesitation sound between segments A and B, while in the other two shows there is a hesitation but no pause. This hesitation or pause happens around the phrase “I like Scooby Doo as well,” which happens after comedian B has established that their aim is to create common ground. The hesitation sound always accompanies this phrase and appears to be an optional pause point. In 2018 Aug 26 there is an additional pause between A and B in which comedian B asks the audience again whether they agree on Scooby Doo as a reference. Every pause ending in usual material has a counterpart in at least one other of the four performances, excluding the pauses in the additions “I’m just saying that” in 2018 Nov 20 and audience interaction in 2018 Aug 25.

The punchline of the joke, occurring after the audience interaction in 2018 Aug 25, has tighter timing of both pauses and speech segments, visible in the closely parallel lines. Without an audience, the delivery of the second section was slower. Managing timing on a larger scale, comedian B ends the Scooby Doo joke sequence early following the additional audience interaction material, effectively making up for time lost during the interaction. In the other two live performances there are two extra pauses with matching phrase endings (“addicts” and “began”) and one additional pause in the lab recording (“began”). Because roughly 20 s of performance time have been used with impromptu audience response on 2018 Aug 25, the last 10 s of the Scooby Doo joke sequence are missed out in the show altogether.

**Fig. 4.**
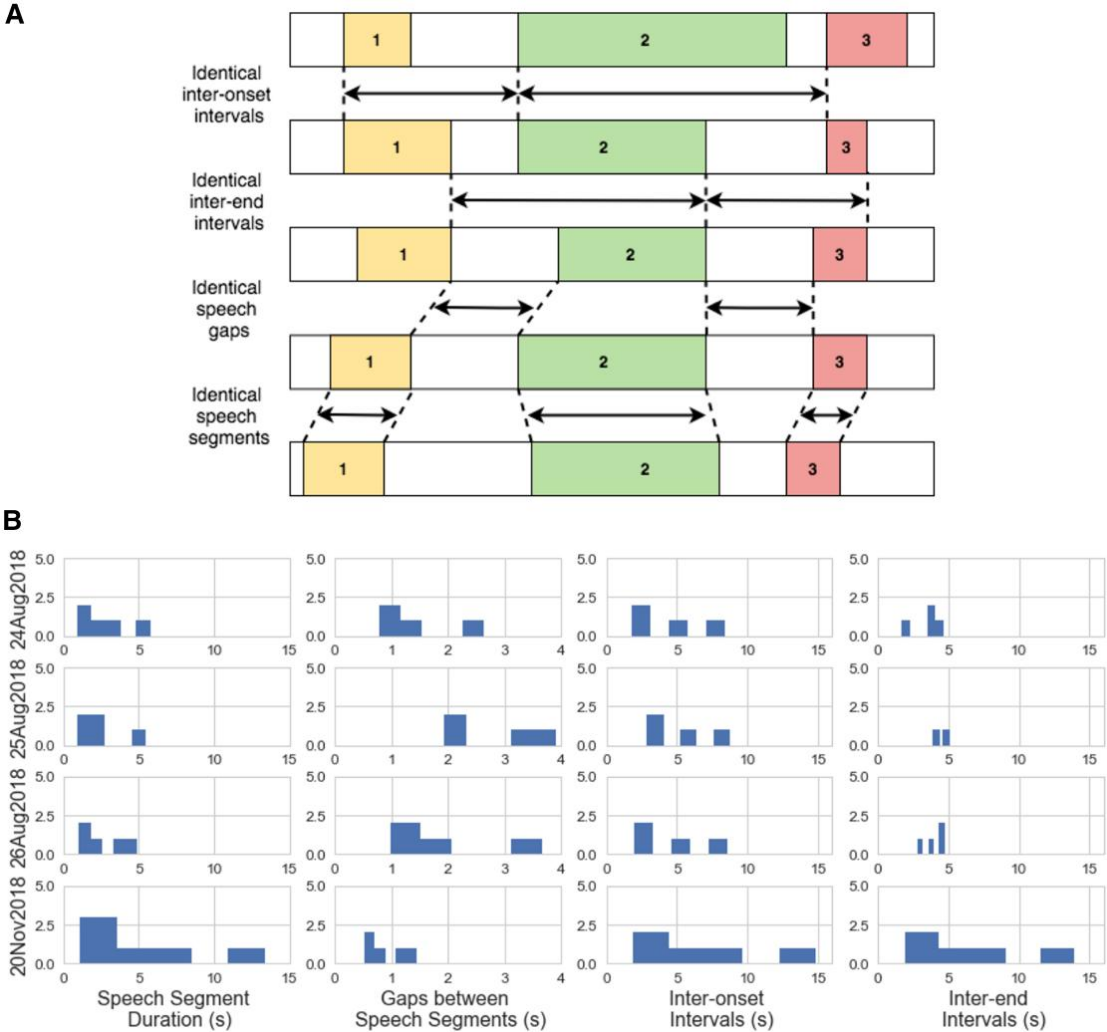
Microtiming parameters and their distribution during comedian B’s punchline. A) Diagram illustrating types of speech segment intervals for microtiming analysis. B) Histograms of microtiming parameters in the four performances of comedian B’s joke in each performance illustrated in Fig. 3B (excluding improvised section in 20Aug2018).

The way speech timing is executed during a punchline informs us about performer priorities in the microtiming of a joke. When repeating exactly the same material in different performances there are several aspects of timing that can be held steady: inter-onset intervals, inter-end intervals, speech gaps and duration of speech segments (Fig. 4A). Although performers probably combine these timing parameters, each one highlights different aspects of speech delivery. Holding inter-onset or inter-end timing steady requires changing gaps between speech and/or the duration of speech. If either the end or onset of speech segments are identical, the joke is being performed as if to an imaginary metronome where the start or end of a segment needs to occur at a particular time in the overall melody of the joke. If speech gaps are identical, the gaps between speech are key to the timing of the joke, perhaps because material elicits reactions of predictable duration at every show. If instead the duration of speech segments holds steady, then the performer is likely telling the same material with similar pacing at the expense of timing between segments.

The duration of speech segments was not significantly different between performances. The extra piece of material in the punchline of 2018 Aug 25 was discounted from this dataset (between sections H and I in Fig. 3B) as there were the unmatched endings. During the performance in the lab, the distribution of speech segment length, inter-onset intervals and inter-end intervals are remarkably similar with only short gaps between speech segments (Fig. 4B). The similarity in the distribution of each of the timing parameters demonstrates the steady delivery used by comedian B in the studio: gaps are regular and similar, as are speech segments, leading to similar inter-onset and inter-end intervals. The three live performances share a similar profile that is distinct from that found in the lab performance. Speech segments are shorter, with more variable gap durations.

The shorter inter-end intervals compared to the inter-onset intervals are due to the first segment of the punchline being a relatively long piece of speech, which is rolled into the first inter-onset interval but not into the first inter-end interval. The distributions of timing parameters in the performances of 2018 August 24, August 25, and August 26 look particularly similar on speech segment duration and inter-onset interval parameters (Fig. 4B). It seems that inter-onset intervals and speech segment duration are the parameters being conserved in the live delivery of the Scooby Doo punchline. The relative pattern of gaps between speech segments is similar, but the actual durations are quite different on 2018 August 25. The longer gap is due to the addition of unmatched material ignored in the punchline dataset and treated as a gap between punchline segments.

## Discussion

Analyzing the way performers do or do not adapt to audiences provides a new avenue into the way live performance can be understood, documented and compared. TAMS, presented in this article, puts the craft of performers’ delivery at the core of the computational method, augmenting existing humanities and digital humanities analyses of performance content, context, ephemera. Crucially, timing and adaptation, both hallmarks of skilled live performance, are leveraged as data that can now be tracked alongside other performance-related data. As well as providing analyses of academic interest, visualizing show variation and stability may be of interest to theater educators, makers, and producers looking to understand their shows better.

By applying scientific methods to multiple performances of a show, we uncovered structural dynamics at both macro- and microscales, in both the material presented and in audience response. The architecture of a show in development becomes visible through the content pillars that remain across 20 performances. A more established show in larger venues had greater consistency in both phrasing and timing with dense sections of exactly repeated sequences chained together in time, forming temporal beams as well as content pillars. Comparing repeated content and audience laughter revealed patterns in silences driven by sequences of content, rather than in highly frequent laughter. The timing of a live joke was robust enough in performance to withstand improvisation immediately before it, but when told in the lab showed a totally different pattern of shortened gaps between speech and more uniform pacing.

TAMS provides a methodology to compare and contrast performances and performers but should not provide the basis for causal inference without additional qualitative and quantitative examinations of additional data types. The case studies of two comedians presented in this article are not sufficient to make any generalizations regarding factors that may explain their unique patterns of matching sequences. The comparable reliability of timing and material in the more experienced performers’ shows could be explained by other factors, some connected to craft, others to context, others to style. For example, the greater temporal stability could be due to the show’s greater maturity, or at another extreme, indicate stagnation. Equally, a more established comedian with larger audiences might create a context in which the audience is more focused and less likely to disrupt the comedian’s prepared material. In terms of microtiming, the disruption to a joke’s timing in the lab could be due to that particular comedian’s lack of experience recording, or be explained by their discomfort with the lab set-up. As performance is also a commercial endeavor, some may be tempted to use performance patterns to optimize shows for financially motivated metrics, such as amount of laughter or target audience demographics. However, the computational methods presented here are not intended to be used to gauge a show’s “success:” a performance is a creative act of communication between creators and audiences.

Drawing attention to performer craft through new methodologies and metrics is intended to open new avenues to documenting, tracking, and analyzing the human ingenuity and processes of live performance. To flatten a performance to only these dimensions is to do a disservice to the nuance and complexity of performance. Similarly, to reduce the value of a performance to positive ratings from the audience or its box office takings is to do a disservice to audiences, as well as performers. Is the “best” comedy show the one in which audiences laughed the most? The one that audiences remembered for longest? The show with the most innovative material? Any one of these possibilities may be a useful metric for a show, but should be guided by sensitive consideration for the creators’ agency and intentions, as well as the research question itself. After all, the most consistently timed audience response across shows was silence, even in the apparently laughter-response-oriented genre of stand-up comedy.

Many performance features can be analyzed with a TAMS approach. The methodology invites scientific researchers to carefully sense the ways in which a particular performance responds to its audience. A Shakespeare play may have word-for-word exact delivery between performances, but the timing between matching sequences may vary, or perhaps the prosody or relative loudness of speech change in varying sequences. Transcripts of multiple productions of the same script could be compared to highlight how shows are abridged, or how different productions extend or shorten particular sections in time depending on their directorial intent. Matching sequences can be identified in nonverbal data, too. Gestures, spatial configurations or positioning on the stage could equally be tokenized, with matching sequences analyzed across several performances. Choreography may be stable over performances, but other factors such as range of movement or microtiming may shift. Live performance demands variability and response, between and within shows.

Live performance is omnipresent across cultures in the form of storytelling, music, dance, ritual, song, theater, puppetry, and clowning, to name a few. Whatever art form it presents in, live performance is embodied, time-bound, responsive to audience and adaptive to context. In this paper, we have shown a series of computational tools that identify common qualitative features of live performance, opening new avenues to understanding this pervasive human behavior. The rise of Artificial Intelligence research and applications has increasingly drawn public awareness to consider whether AI can create art. As AI discourse collapses the value of artistry to scores on esthetic criteria or its plausibility as human output, it is all the more urgent to provide scientific methodologies that explore the variability and diversity of artistic process, expression and delivery. Introducing time dimensions transforms a performance from a static collection of features to one expression of a dynamic system, crafted for human expression and communication.

## Materials and methods

### Materials

Audio of two professional stand-up comedians was collected in United Kingdom venues in 2017 and 2018. Times-stamped transcripts were generated from audio, then manually corrected to ensure disfluencies—nonwords in speech, such as “umm” or “uh”—were included in the resulting files. Details of comedian selection, data gathering, data preprocessing, and code implementation can be found in Supplementary Information and on FigShare. Ethical approval for the study was provided by Queen Mary University of London Ethics Committee (ethics approval: QMREC1954a—Timing in Stand-Up Comedy) and both comedians provided informed consent to take part in the study.

### Algorithm for locating matching sequences

The Python-coded search first finds the longest contiguous sequence common to two sequences, that is a sequence of tokens that has identical content, identical order and has no insertions or deletions. In this research, performance transcripts and performance source text were the content on which the search for matching sequences operated. Once the longest contiguous matching sequence is found, the code then repeats the search process recursively on either side of the match, allowing for matches across segments. If the segments on either side of the matched sequences are (A1, A2) and (B1, B2), respectively, then the next matches are performed on (A1, B1), (A1, B2), (A2, B1), and (A2, B2). The cross-matching catches any material that may not have been presented in sequential order. However, it can also introduce overlaps. An audio clip is created for each matching sequence in each show based on timestamps in the transcript. The search for the longest contiguous sequence continues until the code reaches a preset minimum number of words. Different minima were tested and a sequence of 6 words was used as it avoided matching based on short regular phrases like “I mean look.”

The Python code produces a comma separated value (csv) file that includes: the sequence that was matched, the three tokens (units of a sequence, in this instance word or speech-like sound) immediately before and after the match, the length of the match, the unique ID generated that points back to the created audio clip, the match’s start and end in the dataframe, the match’s start and end in the original audio, and whether the match was sequential or from cross-matching (i.e. A1, B2 or A2, B1).

Interactive visualizations were generated using Processing, static visualizations using Python (further details in Supplementary Information and in Data Availability section).

## Supplementary Material

pgaf394_Supplementary_Data

## Data Availability

Summary data files derived from performer transcripts by TAMS and through annotation are publicly available, along with code to generate TAMS visualizations (40). To protect the anonymity of participating comedians, TAMS .csv file columns that contain content from matching sequences have been removed. TAMS code for extracting matching sequences from two or more transcripts and creating audio clips for each match, along with code for generating Processing visualizations, is available on gitHub and FigShare. Because the original audio and transcripts could not be shared without compromising performer anonymity, an example dataset of publicly available audio files and transcripts to test TAMS code have been available on FigShare (40).
