# Supplementary material for: Timing structures in live comedy: A matched-sequence approach to mapping performance dynamics
Source: PNAS Nexus. 2026 Jan 20;5(1):pgaf394. doi: 10.1093/pnasnexus/pgaf394 (PMC12817072; doi:10.1093/pnasnexus/pgaf394)
Supplement: pgaf394_Supplementary_Data [file pgaf394_supplementary_data.pdf]

Supporting Information for

Timing Structures in Live Comedy: A matched-sequence approach to mapping performance dynamics

Vanessa C. Pope, Rebecca Stewart and Elaine Chew

Vanessa C. Pope
E-mail: vanessa.pope@kcl.ac.uk

This PDF file includes:

Supporting text
Figs. S1 to S2

Supporting Information Text

Materials

Comedian Selection. Two performers were selected to take part in this research. At the time of recording, both were professional comedians, earning a living through writing and performing comedy. The first performer, Comedian A, was an established professional comedian on a medium-scale tour playing to large audience of 150+. The second performer, Comedian B, was an emerging comedian, also a full-time professional, preparing for and attending the Edinburgh Festival Fringe, an annual three-week performing arts festival in Edinburgh, Scotland. It is one of the largest festivals in the world: in 2018 it hosted 3,548 shows in over 300 venues. Comedian B's maximum audience size for recorded shows was 94. Multiple performances from both performers were gathered. Five performances of Comedian A's show were recorded over two months in different venues, while 20 performances of Comedian B's show were gathered over eight months in two venues and in the lab. Comedian A's show had been through Edinburgh and then on tour for several months before recordings were made, whereas Comedian B's show was recorded from its early stages of development. Both performers consented to recordings being gathered for research purposes (ethics approval: QMREC1954a – Timing in Stand-Up Comedy). Comedian B was recruited in January 2018 to take part in a longer case-study of developing work. Comedian B was put forward to be a part of this research by the comedy programmer of an established venue at the Edinburgh Fringe Festival.

Performances of Comedian B's one-hour show were recorded from February to July 2018 as material was workshopped in London for the show's premiere at the Edinburgh Fringe Festival in August 2018. First author VP recorded five performances at the festival, August 11-15 2018 and took notes. The technician for the show then recorded performances from August 17-26 2018 without supervision. All four London performances were recorded in the small performance space in London, which seats roughly 50 people. Recordings were made from the back of the auditorium. The Edinburgh performances were recorded at a venue that seats 94 people. In Edinburgh, the sound desk from which the recordings were made is in the stage right corner of the auditorium facing the stage side-on.

The Comedian B shows analysed in full this research are: Show 1 (3 Feb 2018, London), Show 4 (25 July 2018, London), Show 5 (11 August 2018, Edinburgh), Show 10 (17 August 2018, Edinburgh) and Show 19 (26 August 2018, Edinburgh). This subset was chosen to capture the widest time range in each category and so contains the first and last show recorded in both London and Edinburgh. As Edinburgh is disproportionately represented in the data, the fifth show chosen was between the first and last in Edinburgh.

Audio Recording. A Zoom H4N (Zoom, Japan) was used to capture two audio files for each half of the show at Comedian A's performances, while two audio files per show were gathered at Comedian B's show as their performance had no interval. One audio file is the feed from the performer's microphone, taken directly from the sound desk. The second is the sound in the room, including more of the audience's laughter and responses. Because the Zoom needed to be plugged into the sound desk in under a minute, recording levels were often adjusted during the performance itself, causing some audio artefacts as the volume buttons are pressed. The audio levels of each recording differ, even within those recordings that are high quality, so any analysis of volume would be flawed, even within the same recording. The variability of audio recording quality and settings also prevents any meaningful audio-based analyses of voice or laughter quality.

Script. Comedian A provided the most recent draft of source text for the show, dated December 2016, 4-6 months prior to the recorded performances. The text file is 8023 words long, and broken into approximately 42 paragraphs. Some phrases are signalled as quoted text with double quotation marks. The source text was not altered in any way before it was analysed, despite containing occasional orthographic errors and typos. The text contains paralinguistic features like "oh," and some words or phrases are capitalised, presumably to indicate emphasis, for example, "Come ON! Just driiiiive!". There are also instances of sentences written with interruptions the way they might be spoken in conversation: "someone has suffered for and-and even in tiny things, like I ran for a bus." There are no indications of accompanying physical actions in the text, aside from at the end of the text where an alternative ending is indicated in note form. From comparing the text and the five performances, it is clear that the structure of the show significantly changed since its writing, though a revised script was not produced or provided by the performer.

Comedian B chose not to share their script while it was in development. The cue cards and laptop notes used until August 2018 were not made available either. As the performer writes the script based on a transcription of one of their performances, comparison with the script may not be as valuable a comparison point as Comedian A's, though how Comedian A did not specify how they generate their script. However, Comedian B gave permission for their bullet point list of topics in the show to be photographed at the laboratory recording in November 2018.

Transcripts. All performances (42-62 minutes in length) were automatically transcribed using the commercial service Happy Scribe (38), which has an interface to easily correct the content and timestamp of transcriptions. Two rounds of manual correction were used to: add hesitation sounds and disfluencies, including word repetitions; unify style choices, such as hyphenation, British English and proper noun spellings; and, adjust timestamp errors.

Happy Scribe returns a .srt file (SubRip Text file) that contains formatted text with the timestamps associated with short phrases, usually used for subtitling audio or video. The timestamped transcripts include hesitation noises and disfluent or non-orthographic speech, such as interrupted sentences or word repetitions (eg. "I love I loved yoga"). Correcting a 40-45 minute audio file took approximately 2.5 to 3 hours. A second round of corrections rectified typing errors and unified style (for

example, hyphenation of words). The grouping of phrasings, and thus the timestamps allocated, was automatically produced by Happy Scribe and manually adjusted when these timestamps were more than two lines long. The timestamp groupings do not necessarily reflect natural phrase groupings. As the timestamps for each word are not provided, the pair of timestamps associated with a word is that of the entire grouping of which it is a part. The average number of words per timestamp for Comedian A is 9.36 and for Comedian B is 5.17.

The aim is for each transcription to be an accurate representation of all the speech-like sounds made by the performer on stage. Where possible, exact pronunciation was included in the transcription, so both "because" and its contraction "cos" were noted, reflecting the difference in speech rhythm and emphasis caused by the use of word contractions. Hesitation sounds – such as "UH," "AH" or "HM" – and onomatopoeic, descriptive sounds used in the show (the sound of kissing and of jungle animals, for example) were also included in the transcript. There were some difficulties in transcribing word-play. One joke relied on a homonym changing its meaning up to three times. In this special case, the homonym was transcribed with the spelling associated with its meaning at that point in the joke.

Word sequence matches can be broken by contractions as well as by differences in word choice. All punctuation, aside from apostrophes, is removed and replaced with white space. Apostrophes are regularly used for contractions (eg. "it's," "I've") and are kept intact to avoid creating inaccurate sequence lengths. Hesitation noises and filled pauses -for example, "UH," "AH," "EH," "HM" etc.— are replaced with the code "HES." Correctly identifying the vowel used in a hesitation sound is difficult and uniformly labeling them the same way removes the risk of incorrect sequence breaks. Onomatopoeic sounds were coded with "ONOM." Because Comedian A used many onomatopeia but Comedian B used very few, they were not analysed separately and treated like any other token. Proper names were checked against their spelling in the source text where possible.

White space is used to place each token (word or speech-like sound) in different rows in a dataframe, where it is paired with the most recent start and end timestamp from the .srt file generated by Happy Scribe. The same process occurs with the source text, but the start and end timestamps are set to zero as there is no associated timing information.

Annotations for Laughter and Direct Address. Audio files were manually annotated for laughter types using ELAN software: Audience Laughter (more than five people laughing together), Babble Laughter (2–5 people distinctly laughing), Solo Laughter (one person laughing), Self-laughter (the performer laughing) and Applause/Cheers (the audience applauding, cheering or verbalising in response to the performer). In this analysis, the category "Direct Address" was added to label the performer demanding a particular response from audience member. These categories are subjective, particularly that of Direct Address, which depends on the annotator's perception of the prosody. The question that opens the joke, a variation on "we can agree on Scooby Doo, though, right?", is always labelled as Direct Address even though in some performances the question sounds more rhetorical. In some performances Comedian B speaks the line with energy and leaves a long pause for the audience to respond and in others Comedian B speaks it quickly and moves on to the next sentence after only a short pause. The first round of annotations was made by a trained external annotator who is also an experienced performer. A second round of annotations was conducted for consistency.

In addition to laughter, annotations for speech and hesitations were added to recordings of the Scooby Doo joke in the last three Edinburgh performances (24, 25 and 26 of August 2018) and the lab performance (20 November 2018). The first round of annotations was made by a trained external annotator who is also an experienced performer. First author VP did a second round of annotations for consistency.

Methods

Visualisations.

Chronological Visualisations. For transcript-to-transcript match .csv files, the chronological visualisation draws matches onto two normalised timelines, one for each paired show, and draws lines between the matches. Because only chronological matches are drawn, only the results of four transcript comparisons are visualised (Show 1/2, Show 2/3, Show 3/4, Show 4/5). Each match is colored green in the first show and blue in the second, allowing two comparisons to be displayed on the same show timeline; in Fig. S1, note that the final show (Show 5) never operates as the first show in a pairing so all the matches are blue, whereas other blue matches are largely overwritten by green matches in the next comparison. Mapping matched sequences across multiple performances reveals how the material is used and how different sections of the show operate. Lines drawn between matching sequences on each show's respective normalised timeline highlight how material moves in performance time between shows. The visualisation highlights where matching sequences might be part of the same section as they appear at consistent intervals in normalised performance time and can be identified by the parallel lines drawn between matched pairs in the visualisation (Fig. 1 in main article).

When a match is clicked on in the interactive visualisation, the highlighted sequence appears in red and the corresponding text, the exactly matched sequence with buffer text either side, is displayed about and below (Fig. S1). The timestamps and unique audio clip ID are also displayed. The user can also zoom in to a section of all the show's timelines by dragging their mouse across a segment of any show's timeline (Fig S1). This produces a non-normalised view of that time segment across all shows. Drawing chronological matches helps clarify how material is purposefully evolving or unexpectedly changing. The non-interactive version of a chronological visualisation does not show timestamps or accompanying text (Figs. 1a and 1c in main text). For text-to-transcript matches, lines are not drawn between performances as transcripts are all matched to the same source material: the script (Fig. S2). This visualisation shows instead when each transcript used sequences that were also present in script. Only one of the two performers used in this research provided a written-out script.

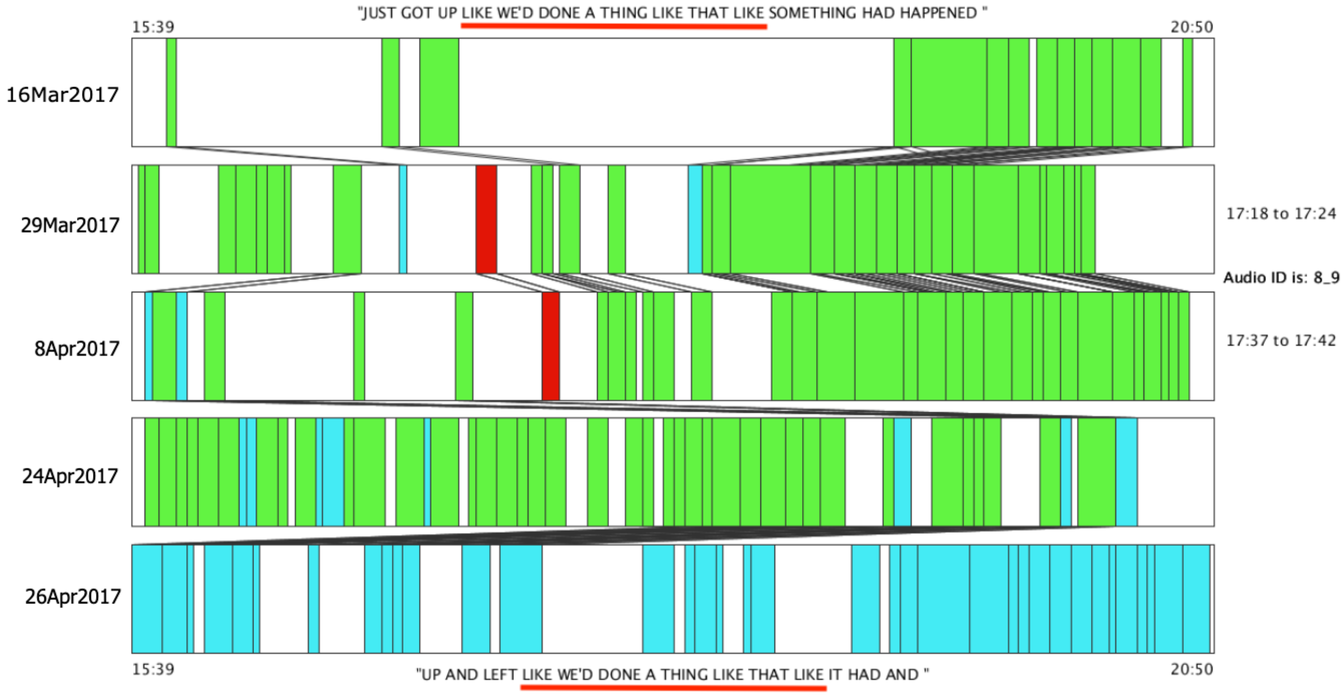

Fig. S1. A zoomed in version of the interactive chronological visualisation and text-performance comparison. The interactive visualisation tool used to zoom into a section of Comedian A's performance time (non-normalised) and to show placement in performance time and the details of each match.

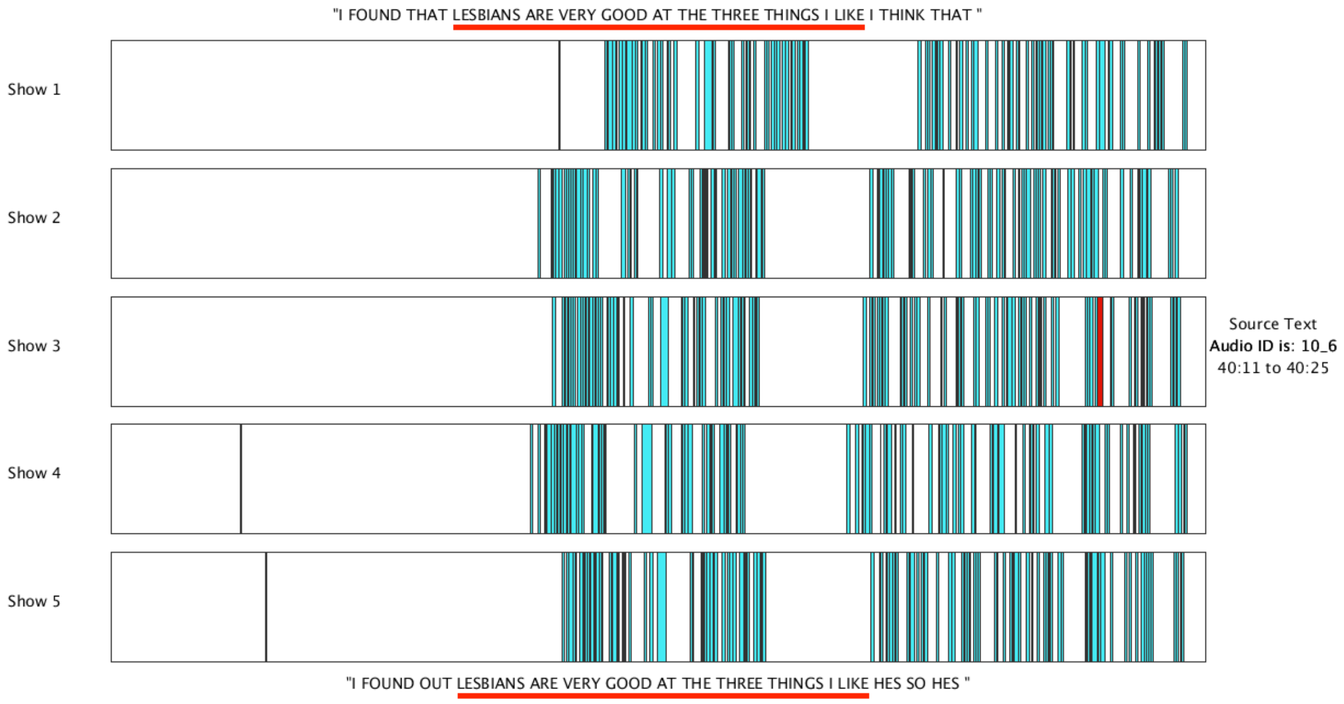

Fig. S2. A zoomed out version of the interactive chronological visualisation that shows placement in performance time of matches to Comedian A 's script in normalised time. This visualisation can also be zoomed into to explore matches in performance time, as in Fig. S1

Genetic Visualisations. The second type of visualisation, referred as "genetic" for its similarity to visualisations of genome sequences, shows how material reoccurs across all pairwise performance comparisons. Fig. 1a and Fig. 1c in the main article show where matching sequences in all performance pairings were located in normalised performance time. Show 1 to 5 are listed top to bottom and represent each show's transcript. Nested within each show's box are five further rows, again representing Shows 1 to 5, to illustrate which matching sequences were found in which other performances. The first row of Show 1 is therefore all black as the whole show matches itself, whereas the third row of Show 3 all black. This visualisation picks out how material recurred between multiple performances, rather than one at a time, but not how material might have moved around show-to-show.

Visualising sequence matches between each transcript and the performer's text illustrates how reliably material from the text appears in the transcripts. Each coloured bar represents a sequence match between the performance transcript and the original text. Its position is in normalised performance time. Only two of the five performances have a matching sequence from text in the first third of the show. Almost halfway through the show there is a series of matches, followed by a gap in all performances where no matching sequences were found. The last third of the performances has another series of matches running up to the very end. There are gaps between the matches, too, which will be interpreted in more detail later in this analysis.

Two main chunks of text are used in performance, while the consistent gap between them suggests that a section of new material has been inserted into all transcripts (Fig. S2). This shape is echoes the matrix of sequence matches between transcripts (Fig. 1a in main text) where matching sequences between one transcript and all others are aligned to give a sense of how material in one performance reoccurs in performances. The beginning of the central column of matches, visible in the text-to-transcript visualisation (Fig. S2) at roughly 0.4–0.5 of performance time, aligns with the dense columns of matching sequences identified in the analysis of transcripts. This section of performance relies on material from the performer's script. Despite the text only accounting for a relatively small proportion of the overall words spoken in performance, it is clear from Fig. S2 that the text is being used most in the second half of the studied performances. In Shows 4 and 5, there is a short match to text at the minimal length of 6 tokens unusually early in the show: "SO WHAT I'M TRYING TO SAY IS." While the excerpt is part of the same joke sequence referencing the audience's freedom to leave the space in both performances, both matches are to an unrelated joke sequence in the source text.
